# Stability of some generalized fractional differential equations in the sense of Ulam–Hyers–Rassias

**DOI:** 10.1186/s13661-023-01695-5

**Published:** 2023-01-26

**Authors:** Abdellatif Ben Makhlouf, El-sayed El-hady, Hassen Arfaoui, Salah Boulaaras, Lassaad Mchiri

**Affiliations:** 1 Mathematics Department, College of Science, Jouf University, P.O. Box: 2014, Sakaka, Saudi Arabia; 2 Department of Mathematics, College of Sciences and Arts, ArRass, Qassim University, Buraydah, Saudi Arabia; 3 Department of Mathematics, Faculty of Sciences of Sfax, University of Sfax, Route Soukra, BP 1171, 3000 Sfax, Tunisia; 4 ENSIIE, University of Evry-Val-d’Essonne, 1 square de la Résistance, 91025 Évry-Courcouronnes Cedex, France

**Keywords:** 47H10, 47J05, Hyers–Ulam–Rassias stability, Generalized fractional differential equation, Fixed-point theory

## Abstract

In this paper, we investigate the existence and uniqueness of fractional differential equations (FDEs) by using the fixed-point theory (FPT). We discuss also the Ulam–Hyers–Rassias (UHR) stability of some generalized FDEs according to some classical mathematical techniques and the FPT. Finally, two illustrative examples are presented to show the validity of our results.

## Introduction

Fractional calculus (FC) has proved to be an efficient tool in the modeling and analysis of many diseases like, e.g., H1N1, COVID-19, and Ebola. This is due to the fact that fractional derivatives can describe the memory and heredity of many processes. Analytical solutions are mainly not reachable for such models (see [1–6]).

Ulam–Hyers stability (UHS) (also known as Ulam stability) for different kind of equations (see [7–10]) plays an essential role as it introduces analytical approximate solutions for many problems where the exact solutions are not reachable. It should be noted that stability is an important issue. This is because if a system is stable in the UHS or UHR sense, then essential properties hold around the exact solution. This can be seen in biology, optimization, and economics (e.g., in particular when an exact solution is quite difficult to obtain). UHS appeared after Ulam’s famous talk at a conference in 1940 (see [7]). Currently, it has become a research trend (see [11]) in many directions.

During the last sixty years, the stability subject has flourished (see [12–21]). In particular, the stability of differential equations (DEs) has attracted the interest of many mathematicians. In 1993, Obloza seems to be the first person who investigated the Ulam stability of DEs (see [22]). In [16], the authors employed the FPT to study the stability of some DEs with delay.

Many authors have studied the UHS for several types of FDEs (see [23–29]). In this sense, our paper presents the existence, uniqueness, and the UHR for a new class of FDEs and generalizes the work in [23].

The article is organized as following. Section 2 recalls some preliminaries, Sect. 3 presents the UHR stability. In Sect. 4, we present a couple of examples to illustrate our results, and Sect. 5 concludes our work.

## Preliminaries

Here, we recall some basic notions and some useful results. Throughout the article, we denote real numbers by $\mathbb{R}$, and the complex numbers by $\mathbb{C}$. We also used the Mittag–Leffler function and generalized metric (see [3, 24, 30]). The theorem of Diaz and Margolis see [31] is the main tool in our analysis.

The objective of the current work is to investigate the stability of the solution of the following generalized FDE

2.1 $$\begin{aligned} dx(\varrho )=f_{1}(\varrho ,x) \,d\varrho +\sum _{i=2}^{n}f_{i}( \varrho ,x) (d \varrho )^{\theta _{i}}, \quad\varrho \in [a,a+b], \theta _{i} \in [0,1], \end{aligned}$$

with $x(a)=x_{0}$, where $a\in \mathbb{R}$, $b>0$ and $x_{0} \in \mathbb{R}$.

### Definition 1

The function $x:[a,a+b]\rightarrow \mathbb{R}$ is named a mild solution of (2.1) if it is a solution of

2.2 $$\begin{aligned} & x(\varrho )=x_{0}+ \int _{a}^{\varrho}f_{1}\bigl(\zeta ,x(\zeta ) \bigr) \,d\zeta + \sum_{i=2}^{n} \theta _{i} \int _{a}^{\varrho}(\varrho -\zeta )^{ \theta _{i}-1} f_{i}\bigl(\zeta ,x(\zeta )\bigr) \,d\zeta , \\ & \quad\varrho \in [a,a+b]. \end{aligned}$$

### Definition 2

Equation (2.2) is UHR stable, if there is a constant $C>0$ such that for each function *y* satisfying

2.3 $$\begin{aligned} \Biggl\vert y(\varrho )-y(a)- \int _{a}^{\varrho}f_{1}\bigl(\zeta ,y(\zeta ) \bigr) \,d \zeta -\sum_{i=2}^{n} \theta _{i} \int _{a}^{\varrho}(\varrho - \zeta )^{\theta _{i}-1} f_{i}\bigl(\zeta ,y(\zeta )\bigr) \,d\zeta \Biggr\vert \leq \epsilon \psi (\varrho ), \end{aligned}$$

∀ $\varrho \in [a,a+b]$, there is a solution $y^{*}(\varrho )$ of (2.2):

$$\begin{aligned} \bigl\vert y(\varrho )-y^{*}(\varrho ) \bigr\vert \leq C \epsilon \psi (\varrho ),\quad \forall \varrho \in [a,a+b]. \end{aligned}$$

### Definition 3

([3])

The Mittag–Leffler function is defined by

$$\begin{aligned} E_{\kappa}(y)= \sum_{m=0}^{+\infty} \frac{y^{m}}{\Gamma (m \kappa +1)}, \end{aligned}$$

where $\kappa >0$, $y\in \mathbb{C}$.

## Stability results

Define $E:=C([a,a+b],\mathbb{R})$. We start with the UHR stability of (2.2).

### Theorem 1

*Let* $L_{f_{i}}>0$, $i\in \{1,2,\ldots,n \}$ *be constants*. *Assume that* $f_{i}:[a,a+b]\times \mathbb{R}\rightarrow \mathbb{R}$, *satisfies*

3.1 $$\begin{aligned} \bigl\vert f_{i}(\varrho ,\sigma _{1})-f_{i}(\varrho ,\sigma _{2}) \bigr\vert \leq L_{f_{i}} \vert \sigma _{1}-\sigma _{2} \vert ,\quad \forall \varrho \in [a,a+b], \sigma _{1}, \sigma _{2}\in \mathbb{R}, i\in \{1,2,\ldots,n \}. \end{aligned}$$

*If a continuous function* $y:[a,a+b]\rightarrow \mathbb{R}$ *satisfies*

3.2 $$\begin{aligned} & \Biggl\vert y(\varrho )-y(a)- \int _{a}^{\varrho}f_{1}\bigl(\eta ,y(\eta ) \bigr) \,d \eta -\sum_{i=2}^{n} \theta _{i} \int _{a}^{\varrho}(\varrho -\eta )^{ \theta _{i}-1} f_{i}\bigl(\eta ,y(\eta )\bigr) \,d\eta \Biggr\vert \leq \epsilon \psi ( \varrho ), \\ &\quad \forall \varrho \in [a,a+b], \end{aligned}$$

*where* $\psi : [a,a+b] \rightarrow \mathbb{R}_{+}$ *is a nondecreasing continuous function*, *then a unique solution* $y^{*}$ *of* (2.2) *exists such that*

$$\begin{aligned} \bigl\vert y(\varrho )-y^{*}(\varrho ) \bigr\vert \leq \frac{e^{(L_{f_{1}}+\delta )T}\prod_{i=2}^{n} \mathbb{E}_{\theta _{i}} ((L_{f_{i}}+\delta )T^{\theta _{i}} )}{1-c} \epsilon \psi (\varrho ), \quad\forall \varrho \in [a,a+b], \end{aligned}$$

*where* $c= ( \frac{L_{f_{1}}}{L_{f_{1}}+\delta}+\sum_{i=2}^{n} \frac{L_{f_{i}}}{L_{f_{i}}+\delta}\Gamma (\theta _{i}+1) ) <1$, $\delta >0$, *and* $\Gamma (\cdot )$ *is the well*-*known Gamma function*.

### Proof

First, we define the following metric on *E*

3.3 $$\begin{aligned} d(x_{1},x_{2}):=\inf \biggl\{ c\geq 0: \frac{ \vert x_{1}(\varrho )-x_{2}(\varrho ) \vert }{\varphi (\varrho )} \leq c \psi (\varrho ), \forall \varrho \in [a,a+b] \biggr\} , \end{aligned}$$

where $\varphi (\varrho ):=e^{(L_{f_{1}}+\delta )(\varrho -a)}\times \prod_{i=2}^{n} \mathbb{E}_{\theta _{i}} ((L_{f_{i}}+\delta )(\varrho -a)^{\theta _{i}} )$. The space $(E,d)$ is a complete generalized metric space.

Let us consider the operator $\mathcal{A}:E \rightarrow E$:

$$\begin{aligned} &(\mathcal{A}u) (\varrho ):=y(a)+ \int _{a}^{\varrho}f_{1}\bigl(\zeta ,u( \zeta ) \bigr) \,d\zeta +\sum_{i=2}^{n} \theta _{i} \int _{a}^{\varrho}( \varrho -\zeta )^{\theta _{i}-1} f_{i}\bigl(\zeta ,u(\zeta )\bigr) \,d\zeta , \\ & \quad\forall t\in [a,a+b]. \end{aligned}$$

Since $\mathcal{A} u \in E$, for every $u\in E$ and

$$\begin{aligned} \frac{ \vert (\mathcal{A}u_{0})(\varrho )-u_{0}(\varrho ) \vert }{\varphi (\varrho )} < + \infty ,\quad \forall u_{0}\in E, \varrho \in [a,a+b], \end{aligned}$$

it is clear that $d(\mathcal{A}u_{0},u_{0}) < \infty $. Moreover, since $d(u_{0},u)< \infty $, $\forall u\in E$, then $\{u\in E: d(u_{0},u)< \infty \}=E$.

In addition, for any $x_{1},x_{2} \in E$ we obtain

3.4 $$\begin{aligned} & \bigl\vert (\mathcal{A} x_{1}) (\varrho )-(\mathcal{A} x_{2}) (\varrho ) \bigr\vert \\ &\quad\leq \biggl| \int _{a}^{\varrho} \bigl[f_{1}\bigl(\zeta ,x_{1}(\zeta )\bigr)-f_{1}( \zeta ,x_{2}(\zeta ) \bigr] \,d\zeta \biggr\vert \\ &\qquad{} + \Biggl| \sum_{i=2}^{n} \theta _{i} \int _{a}^{\varrho} (\varrho - \zeta )^{\theta _{i}-1} \bigl[f_{i}\bigl(\zeta ,x_{1}(\zeta )\bigr)-f_{i}( \zeta ,x_{2}( \zeta )\bigr] \,d\zeta \Biggr|. \end{aligned}$$

Then, we derive that

3.5 $$\begin{aligned} & \bigl\vert (\mathcal{A} x_{1}) (\varrho )-(\mathcal{A} x_{2}) (\varrho ) \bigr\vert \\ &\quad\leq \int _{a}^{\varrho} \bigl\vert f_{1}\bigl( \zeta ,x_{1}(\zeta )\bigr)-f_{1}\bigl( \zeta ,x_{2}(\zeta )\bigr) \bigr\vert \,d\zeta \\ &\qquad{}+\sum_{i=2}^{n} \theta _{i} \int _{a}^{\varrho} (\varrho -\zeta )^{ \theta _{i}-1} \bigl\vert f_{i}\bigl(\zeta ,x_{1}(\zeta )\bigr)-f_{i} \bigl(\zeta ,x_{2}( \zeta )\bigr) \bigr\vert \,d\zeta \\ &\quad\leq L_{f_{1}} \int _{a}^{\varrho} \bigl\vert x_{1}(\zeta )-x_{2}(\zeta ) \bigr\vert \,d\zeta +\sum _{i=2}^{n} \theta _{i} L_{f_{i}} \int _{a}^{ \varrho} (\varrho -\zeta )^{\theta _{i}-1} \bigl\vert x_{1}(\zeta )-x_{2}( \zeta ) \bigr\vert \,d\zeta \\ &\quad\leq L_{f_{1}} \int _{a}^{\varrho} \frac{ \vert x_{1}(\zeta )-x_{2}(\zeta ) \vert e^{(L_{f_{1}}+\delta )(\zeta -a)} \prod_{i=2}^{n}\mathbb{E}_{\theta _{i}} ((L_{f_{i}}+\delta )(\zeta -a)^{\theta _{i}} ) \,d\zeta }{e^{(L_{f_{1}}+\delta )(\zeta -a)}\prod_{i=2}^{n} \mathbb{E}_{\theta _{i}} ((L_{f_{i}}+\delta )(\zeta -a)^{\theta _{i}} )} \\ &\qquad{}+\sum_{i=2}^{n} \theta _{i} L_{f_{i}} \int _{a}^{\varrho} \frac{(\varrho -\zeta )^{\theta _{i}-1} \vert x_{1}(\zeta )-x_{2}(\zeta ) \vert e^{(L_{f_{1}}+\delta )(\zeta -a)}\prod_{i=2}^{n}\mathbb{E}_{\theta _{i}} ((L_{f_{i}}+\delta )(\zeta -a)^{\theta _{i}} )}{e^{(L_{f_{1}}+\delta )(\zeta -a)} \prod_{i=2}^{n}\mathbb{E}_{\theta _{i}} ((L_{f_{i}}+\delta )(\zeta -a)^{\theta _{i}} )} \,d\zeta \\ &\quad\leq d(x_{1},x_{2}) \Biggl[L_{f_{1}} \int _{a}^{\varrho} \psi ( \zeta )e^{(L_{f_{1}}+\delta )(\zeta -a)} \,d \zeta \prod_{i=2}^{n} \mathbb{E}_{\theta _{i}} \bigl((L_{f_{i}}+\delta ) (\varrho -a)^{\theta _{i}} \bigr) \\ &\qquad{}+e^{(L_{f_{1}}+\delta )(\varrho -a)}\sum_{i=2}^{n} \theta _{i} L_{f_{i}} \int _{a}^{\varrho}\psi (\zeta ) (\varrho -\zeta )^{\theta _{i}-1} \prod_{i=2}^{n} \mathbb{E}_{\theta _{i}} \bigl((L_{f_{i}}+\delta ) ( \zeta -a)^{\theta _{i}} \bigr) \,d\zeta \Biggr], \end{aligned}$$

which can easily be rewritten as

3.6 $$\begin{aligned} \bigl\vert (\mathcal{A} x_{1}) (\varrho )-( \mathcal{A} x_{2}) (\varrho ) \bigr\vert & \leq d(x_{1},x_{2}) \biggl[ \frac{L_{f_{1}} \psi (\varrho )\varphi (\varrho ) }{L_{f_{1}}+\delta}+ \frac{\sum_{i=2}^{n}\theta _{i} L_{f_{i}} \Gamma (\theta _{i})}{L_{f_{i}}+\delta} \psi (\varrho ) \varphi (\varrho ) \biggr] \\ &\leq \Biggl(\frac{L_{f_{1}} }{L_{f_{1}}+\delta}+ \sum_{i=2}^{n} \frac{L_{f_{i}} \Gamma (\theta _{i}+1)}{L_{f_{i}}+\delta} \Biggr)\,d(x_{1},x_{2}) \varphi (\varrho ) \psi (\varrho ). \end{aligned}$$

Therefore,

$$\begin{aligned} d(\mathcal{A} x_{1},\mathcal{A} x_{2})\leq c d(x_{1},x_{2}), \end{aligned}$$

which proves that $\mathcal{A}$ is strictly contractive. From (3.6) it follows that

$$\begin{aligned} d(y,\mathcal{A}y)\leq \epsilon . \end{aligned}$$

Now, as a consequence of the Diaz and Margolis Theorem (see [31]), there exists a solution $y^{*}$:

$$\begin{aligned} d\bigl(y^{*},y\bigr)\leq \frac{1}{1-c} \epsilon \end{aligned}$$

and then

$$\begin{aligned} \bigl\vert y^{*}(\varrho )-y(\varrho ) \bigr\vert \leq \frac{\epsilon}{1-c} \varphi ( \varrho ) \psi (\varrho ), \end{aligned}$$

for all $t\in [a,a+b]$, which implies that

$$\begin{aligned} \bigl\vert y^{*}(\varrho )-y(\varrho ) \bigr\vert \leq \frac{ e^{(L_{f_{1}}+\delta )(\varrho -a)} \prod_{i=2}^{n}\mathbb{E}_{\theta _{i}} ((L_{f_{i}}+\delta )(\varrho -a)^{\theta _{i}} )}{1-c} \epsilon \psi (\varrho ), \end{aligned}$$

for all $\varrho \in [a,a+b]$. □

### Remark 1

It should be noted that when $f_{1}=0$, $f_{i}=0,i\ge 3$ we easily obtain the results in [23] and when $f_{i}=0$, $i\geq 2$ we obtain the results in [32].

The next theorem is a direct consequence of Theorem 1 (Ulam stability of (2.2)).

### Theorem 2

*Let* $L_{f_{i}}>0$, $i\in \{1,2,\ldots,n \}$ *be constants*. *Assume that* $f_{i}:[a,a+b]\times \mathbb{R}\rightarrow \mathbb{R}$, *satisfies*

3.7 $$\begin{aligned} \bigl\vert f_{i}(\varrho ,\sigma _{1})-f_{i}(\varrho ,\sigma _{2}) \bigr\vert \leq L_{f_{i}} \vert \sigma _{1}-\sigma _{2} \vert ,\quad \forall \varrho \in [a,a+b], \sigma _{1}, \sigma _{2}\in \mathbb{R}, i\in \{1,2,\ldots,n \}. \end{aligned}$$

*If a continuous function* $y:[a,a+b]\rightarrow \mathbb{R}$ *satisfies*

3.8 $$\begin{aligned} &\Biggl\vert y(\varrho )-y(a)- \int _{a}^{\varrho}f_{1}\bigl(\zeta ,y(\zeta ) \bigr) \,d \zeta -\sum_{i=2}^{n}\theta _{i} \int _{a}^{\varrho}(\varrho -\zeta )^{ \theta _{i}-1} f_{i}\bigl(\zeta ,y(\zeta )\bigr) \,d\zeta \Biggr\vert \leq \epsilon , \\ & \quad\forall \varrho \in [a,a+b], \end{aligned}$$

*then a unique solution* $y^{*}$ *of* (2.2) *exists satisfying*

$$\begin{aligned} \bigl\vert y(\varrho )-y^{*}(\varrho ) \bigr\vert \leq \frac{e^{(L_{f_{1}}+\delta )T} \prod_{i=2}^{n} \mathbb{E}_{\theta _{i}} ((L_{f_{i}}+\delta )T^{\theta _{i}} )}{1-c} \epsilon ,\quad \forall \varrho \in [a,a+b], \end{aligned}$$

*where* $c= ( \frac{L_{f_{1}}}{L_{f_{1}}+\delta}+\sum_{i=2}^{n} \frac{L_{f_{i}}}{L_{f_{i}}+\delta}\Gamma (\theta _{i}+1) )<1$, $\delta >0$, *and* $\Gamma (\cdot )$ *is the well*-*known Gamma function*.

## Examples

A couple of examples are used to show the validity of Theorem 1 and Theorem 2.

### Example 1

Let (2.1) for ${\theta}=0.5$, $a=0$, $b=2$, $f_{1}(\alpha ,\beta )=\alpha ^{2} \sin (\beta )$, $f_{2}(\alpha ,\beta )=\alpha \cos (\beta )$ and $f_{i}=0, i\in \{3,4,\ldots,n \}$.

We have

$$\begin{aligned} \bigl\vert \alpha ^{2}\sin (\beta _{1})- \alpha ^{2}\sin (\beta _{2}) \bigr\vert \leq 4 \vert \beta _{1}-\beta _{2} \vert ,\quad \forall \alpha \in [0,2], \beta _{1}, \beta _{2}\in \mathbb{R}, \end{aligned}$$

and

$$\begin{aligned} \bigl\vert \alpha \cos (\beta _{1})- \alpha \cos (\beta _{2}) \bigr\vert \leq 2 \vert \beta _{1}- \beta _{2} \vert ,\quad \forall \alpha \in [0,2], \beta _{1}, \beta _{2} \in \mathbb{R}. \end{aligned}$$

Then, $L_{f_{1}}=4$ and $L_{f_{2}}=2$.

Suppose that *y* satisfies

4.1 $$\begin{aligned} \biggl\vert y(\varrho )-y(0)- \int _{0}^{\varrho} s^{2} \sin \bigl(y(s) \bigr) \,ds-0.5 \int _{0}^{\varrho}(\varrho -s)^{-0.5} s \cos \bigl( y(s) \bigr) \,ds \biggr\vert \leq \varrho , \end{aligned}$$

for all $\varrho \in [0,2]$.

Here, $\epsilon =1$ and $\psi (\varrho )=\varrho $. In view of Theorem 1 there is a continuous function $y^{*}$,

$$\begin{aligned} y^{*}(\varrho )=y(0)+ \int _{0}^{\varrho}s^{2} \sin \bigl(y^{*}(s) \bigr) \,ds+0.5 \int _{0}^{\varrho}(\varrho -s)^{-0.5} s \cos \bigl( y^{*}(s) \bigr) \,ds, \end{aligned}$$

such that

$$\begin{aligned} \bigl\vert y(\varrho )-y^{*}(\varrho ) \bigr\vert \leq \frac{ e^{16} \mathbb{E}_{0.5}(6 \sqrt{2})}{1- (\frac{1}{2}+\frac{1}{3} \Gamma (1.5) )} \varrho , \quad\forall \varrho \in [0,2]. \end{aligned}$$

### Example 2

Let equation (2.1) for ${\theta}=0.6$, $a=0$, $b=5$, $f_{1}(\alpha ,\beta )=\alpha \cos (\beta )$, $f_{2}(\alpha ,\beta )= \sin (\beta )$ and $f_{i}=0, i\in \{3,4,\ldots,n \}$.

We have

$$\begin{aligned} \bigl\vert \alpha \cos (\beta _{1})- \alpha \cos (\beta _{2}) \bigr\vert \leq 5 \vert \beta _{1}- \beta _{2} \vert ,\quad \forall \alpha \in [0,5], \beta _{1}, \beta _{2} \in \mathbb{R}, \end{aligned}$$

and

$$\begin{aligned} \bigl\vert \sin (\beta _{1})- \sin (\beta _{2}) \bigr\vert \leq \vert \beta _{1}-\beta _{2} \vert ,\quad \forall \alpha \in [0,5], \beta _{1}, \beta _{2}\in \mathbb{R}. \end{aligned}$$

Then, $L_{f_{1}}=5$ and $L_{f_{2}}=1$.

Suppose that *y* satisfies

4.2 $$\begin{aligned} \biggl\vert y(\varrho )-y(0)- \int _{0}^{\varrho} s \cos \bigl(y(s) \bigr) \,ds-0.6 \int _{0}^{\varrho}(\varrho -s)^{-0.4} \sin \bigl( y(s) \bigr) \,ds \biggr\vert \leq 0.1, \end{aligned}$$

for all $\varrho \in [0,5]$.

Here, $\epsilon =0.1$. Employing Theorem 2 there is a continuous function $y^{*}$,

$$\begin{aligned} y^{*}(\varrho )=y(0)+ \int _{0}^{\varrho}s \cos \bigl(y^{*}(s) \bigr) \,ds+0.6 \int _{0}^{\varrho}(\varrho -s)^{-0.4} \sin \bigl( y^{*}(s) \bigr) \,ds, \end{aligned}$$

such that

$$\begin{aligned} \bigl\vert y(\varrho )-y^{*}(\varrho ) \bigr\vert \leq \frac{ e^{50} \mathbb{E}_{0.6}(6 \times 5^{0.6})}{1- (\frac{1}{2}+\frac{1}{6} \Gamma (1.6) )} 0.1,\quad \forall \varrho \in [0,5]. \end{aligned}$$

## Conclusion

In this paper, we utilized some results of Banach FPT to study the existence, uniqueness, and the UHR stability of some generalized FDEs. Finally, we have presented two examples to illustrate our results. In future work, we intend to extend our results to the stochastic case.

## Data Availability

Not applicable.
